# Educational Interventions for Chronic Obstructive Pulmonary Disease (COPD) in Care Homes: A Near-Empty Scoping Review Revealing a Major Evidence Gap

**DOI:** 10.3390/nursrep16020043

**Published:** 2026-01-29

**Authors:** Bronach Campbell, Gary Mitchell, Stephanie Craig, Tara Anderson

**Affiliations:** School of Nursing and Midwifery, Queen’s University Belfast, Belfast BT9 7BL, UK; bcampbell25@qub.ac.uk (B.C.); gary.mitchell@qub.ac.uk (G.M.); s.craig@qub.ac.uk (S.C.)

**Keywords:** Chronic Obstructive Pulmonary Disease, education, staff training, care homes, nursing homes, care delivery, scoping review

## Abstract

**Background/Objectives:** Chronic Obstructive Pulmonary Disease (COPD) is highly prevalent among individuals residing in care homes, where effective disease management can enhance quality of life by slowing disease progression. Care home staff are central to COPD management in these settings, and their capacity to deliver optimal care may be strengthened through targeted education and training interventions. This scoping review aimed to synthesise existing evidence on education and training intended to enhance COPD care delivery by care home staff. **Methods:** A scoping review was conducted in accordance with Joanna Briggs Institute (JBI) guidelines and reported in line with the Preferred Reporting Items for Systematic Reviews and Meta-Analyses extension for Scoping Reviews (PRISMA-ScR) framework. Four electronic databases (CINAHL, EMBASE, MEDLINE, and PsycINFO) were systematically searched for studies evaluating educational or training interventions regarding COPD for care home staff. **Results:** Only one study met the eligibility criteria for inclusion. This mixed methods study encompassed both a randomised control trial and semi-structured interviews, evaluating the effects of a COPD education programme for healthcare professionals working in a care home setting. This education intervention led to increased COPD-related knowledge and improved support for staff managing residents with COPD. **Conclusions:** Evidence for educational interventions for care home staff caring for individuals with COPD is extremely limited. While the included study shows potential for educational programmes, substantial gaps persist. Further research is needed to develop, implement, and rigorously assess education and training interventions to support high-quality COPD care in care homes.

## 1. Introduction

Chronic Obstructive Pulmonary Disease (COPD) is a progressive lung disease characterised by obstruction of airflow in the lungs and is a common cause of death globally [1,2,3]. COPD symptoms can present as tightness of chest, wheezing, breathlessness (dyspnea), chronic cough and sputum production, with varying levels of severity [2,3]. Multiple cells play a role in COPD pathophysiology, including macrophages, epithelial cells, and T-lymphocytes [4]. Substance exposure causes dysfunction of these cells, resulting in elevated production of inflammatory mediators and therefore furthering disease progression [4]. As the disease progresses, airflow is reduced and gas exchange becomes increasingly impaired, leading to degradation of lung tissue and, ultimately, the development of chronic respiratory failure. This often results in dependency on long-term oxygen therapy [5].

COPD exacerbations are associated with physical decline and worsening symptoms, such as shortness of breath, increased sputum production, cough, and sore throat [2,6,7]. These events are believed to result from a combination of respiratory viruses and bacteria, individual susceptibility, and environmental pollutants, leading to an increased inflammatory burden [8]. The frequency of exacerbations can vary between patients, but those who experience them more often tend to have a poorer quality of life [6,9].

There is limited data regarding prevalence within care homes, although one retrospective analysis of data in the United States of America found 21.5% of residents with cognitive impairment had a diagnosis of COPD [10]. Whilst the exact figures are undetermined, COPD is prevalent within care homes [10,11]. COPD exacerbations can result in prolonged recovery periods, with some individuals becoming housebound and dependent on others [12]. The disease is also associated with reduced physical activity and a progressive decline in quality of life (QoL) [6,12]. Care home residents experience higher dependency on others to meet their daily living needs, by virtue of their health conditions and medical history [13]. Care home residents are more likely to be frail and have an increased risk of acute hospitalisations [14], similarly to those with COPD. Therefore, it can be inferred that prevalence in care homes may be high due to disease implications.

Optimal COPD care requires attention to nutrition and physical functioning, in addition to pharmacological management [15,16]. Malnutrition and low muscle mass are common in COPD and are associated with poorer lung function, increased exacerbations, reduced exercise tolerance, and higher mortality [17,18]. Nutritional support and pulmonary rehabilitation programmes, including exercise training and early rehabilitation after exacerbations, can improve nutritional status, functional capacity, and health-related quality of life, particularly when nutrition therapy is combined with rehabilitation [16,19,20,21,22]. However, these multidisciplinary approaches have been largely developed in hospital and community settings, and there is little evidence on how care home staff are prepared, through structured education and training, to recognise and respond to malnutrition, functional decline, and rehabilitation needs in residents with COPD.

The nurse role can enable effective management of COPD as nurses are often the initial point of contact for patients and are involved in care throughout every stage of life [23,24]. The NHS in England has planned to invest in further training for all staff, and a new desirable criterion for senior staff is an MSc qualification relevant to their role [25]. However, whilst healthcare providers offer education tools, a significant percentage of staff do not complete professional development courses following their original training [26]. This suggests difficulty in ensuring nurse education post-qualification as training is often optional and based on individual interest. Further training is, however, a mandatory part of the Nursing Midwifery Council (NMC) revalidation process, but it does not specify which training [27]. Perhaps in-depth COPD education should be an essential component of undergraduate training for all nurses, ensuring they are equipped with knowledge of the disease prior to qualifying. The NMC and university stakeholders ensure undergraduate education is relevant to contemporary health [28], but specific teachings are not regulated.

The need for education and training in care homes regarding both pharmacological and non-pharmacological interventions for COPD is clear. Although the evidence is dated, early diagnosis and intervention have been shown to slow disease progression and improve residents’ QoL, with the potential for a partial reversal of the condition [29,30]. Supporting care home staff to care for COPD residents can also enhance residents’ holistic well-being [31]. However, previous research implies those in primary care lack sufficient knowledge to provide optimal care for those with COPD when compared to hospital staff [32,33]. For example, nurses and other healthcare professionals have demonstrated a lack of competence in inhaler technique for those with COPD [34,35]. This lack of knowledge is highlighted by the fact that adults newly admitted to care homes, from hospitals, have a high acute re-admission rate when COPD is part of their medical history [36].

Care homes’ statements of purpose for categories of care vary throughout the sector; however, whether it is dementia care, or care of an older adult, COPD can be a diagnosis for many of these residents. One example of educational interventions improving patient outcomes in care homes is a training programme on care for people with dementia, which found challenging behaviour decreased significantly three months post-training [37]. Another scoping review found that, although resident outcomes did not improve, interprofessional communication and staff ability to conduct heart failure assessments did improve post-educational intervention; moreover, nurses expressed appreciation for the additional training [38]. This supports the idea that further educational interventions can improve practice and nurse confidence in the management of long-term health conditions and therefore may be beneficial within the context of COPD.

The present scoping review, therefore, aimed to map and describe the extent, nature, and characteristics of evidence on education and training interventions for care home staff regarding COPD, and to identify gaps to inform future research and practice. A scoping review method was selected because the primary aim was not to evaluate the effectiveness of a well-established body of interventions, but to systematically map any existing COPD-related education and training for care home staff. Current methodological guidance indicates that scoping reviews are particularly appropriate when the evidence base is expected to be heterogeneous, emergent, or sparse, and when clarifying what has and has not yet been studied [39]. Using a scoping review therefore ensured a structured systematic approach capable of accommodating an anticipated paucity of evidence while still producing an informative evidence map.

Objectives:

1. To identify and map published studies that report education or training interventions on COPD delivered to staff working in care home settings.
2. To describe the key characteristics and reported outcomes of COPD-related education and training interventions for care home staff.
3. To identify gaps in the existing evidence on COPD education and training for care home staff, in order to highlight priorities for future intervention development and evaluation.

Research Question: What COPD-related education and training interventions for care home staff have been evaluated in the published literature?

## 2. Materials and Methods

### 2.1. Protocol and Registration

This scoping review was conducted in line with the Joanna Briggs Institute (JBI) methodology for scoping reviews [40] and was reported in accordance with the Preferred Reporting Items for Systematic Reviews and Meta-Analyses extension for Scoping Reviews (PRISMA-ScR) checklist [41]. A protocol for this scoping review was registered prospectively on Open Science Framework on 16 April 2025 (registration DOI: 10.17605/OSF.IO/VA5PF).

### 2.2. Eligibility Criteria

#### 2.2.1. Types of Sources

This scoping review considered both experimental and quasi-experimental study designs including randomised controlled trials, non-randomised controlled trials, before-and-after studies, and interrupted time-series studies. Analytical observational studies, including prospective and retrospective cohort studies, case–control studies, and analytical cross-sectional studies, were considered for inclusion. This review also considered descriptive observational study designs including case series, individual case reports, and descriptive cross-sectional studies for inclusion. Studies were also considered that focused on qualitative data, including phenomenology, grounded theory, ethnography, and qualitative description. Identified systematic reviews were searched for relevant papers found in their reference sections that may not have been found in the database searches. Studies published in English only were included due to a lack of language diversity amongst the researchers. No date restrictions were applied, and grey literature was not included to maintain rigour and reliability by focusing on peer-reviewed publications. Further eligibility criteria were developed using the Population, Concept, and Context (PCC) framework, as recommended by JBI [42].

##### Population

The population of interest was care home staff. No restrictions were placed on care home type, participant gender, age, or ethnicity. Studies were considered for inclusion if at least some of the sample consisted of care home staff, for example, those which included residents.

##### Concept

All education and training focused on COPD were considered for inclusion. This may have included education related to early diagnosis, assessment, or management. Studies focused primarily on other respiratory illnesses were excluded. Studies which evaluated outcomes such as staff knowledge, confidence, and self-efficacy, as well as resident outcomes, such as improved quality of life, were considered. Additionally, studies which reported on staff experiences of engaging with an education or training intervention on COPD were considered.

##### Context

No restrictions were placed on geographic location. All studies which reported educational or training interventions in a care home setting were included. Care home environments included nursing homes, residential care homes, supported living settings, residential aged care settings, and other long-term care settings. Long-term hospital wards, rehabilitation units, and skilled nursing facilities were not included due to the varying care structures, goals, and patient populations compared to care home settings.

### 2.3. Search Strategy

The search strategy was developed iteratively by the review team in close collaboration with an experienced health-sciences subject librarian. The librarian advised on both the formulation of the research question and the operationalisation of the PCC elements into database search terms. An initial limited search of Google Scholar was conducted to identify key articles and index terms related to COPD education and training in care home settings, and these findings were used to inform the search strategy. A comprehensive search strategy was then constructed for MEDLINE (Supplementary Material 2) and systematically adapted to the syntax and subject headings of each database searched (CINAHL, EMBASE, MEDLINE, and PsycINFO). To identify potentially relevant studies, the bibliographic databases were searched on 22 April 2025. To enhance completeness, the reference list and citations of the included study were also screened for additional eligible articles.

### 2.4. Selection of Sources of Evidence

Following the search, all identified citations were collated and uploaded into Covidence systematic review software, Veritas Health Innovation, Melbourne, Australia, available at https://www.covidence.org/ (accessed on 22 April 2025), a screening and data extraction tool for streamlining the production of reviews. Following the removal of duplicates, BC carried out full screening of the title and abstracts; GM and TA independently repeated this for 50% of the records each. The same procedure was used for full-text screening. Disagreements throughout the screening process were resolved following a discussion between authors.

### 2.5. Data Charting

Charting the data was carried out in Covidence, guided by the ‘JBI template source for evidence details, characteristics and results extraction instrument’ [43]. BC conducted data extraction from the paper included in the scoping review, and this was checked for accuracy by TA independently. The data extracted included specific details about the author, year, setting, participants, study methods, outcomes, and key findings.

In accordance with JBI guidance for scoping reviews, a formal quality appraisal of the included studies was not undertaken. The purpose of this review was to chart the evidence related to COPD-education for care home staff in order to identify knowledge gaps and priority areas for future education and practice, rather than to assess the methodological quality of existing evidence [40,41].

## 3. Results

**Figure 1:**
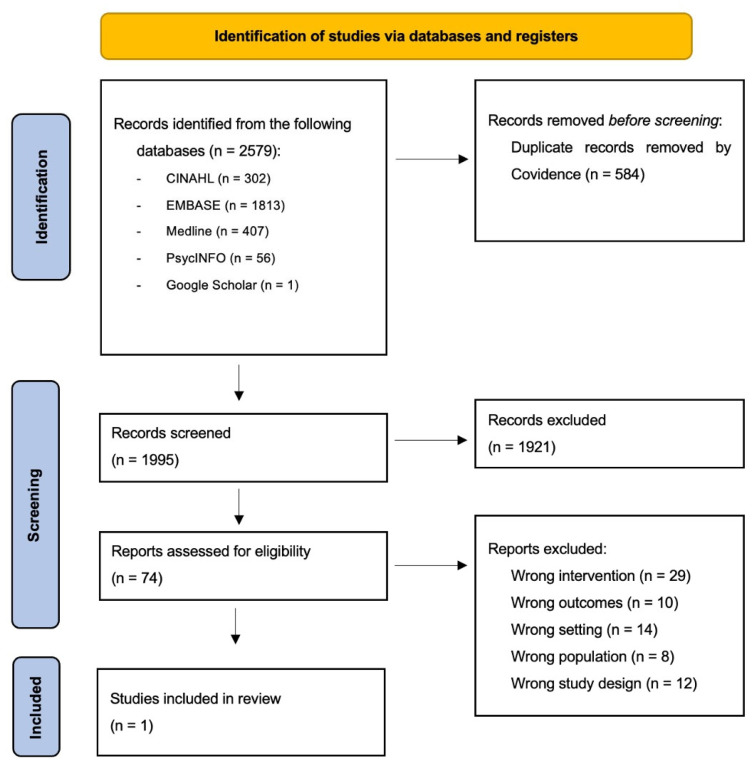
PRISMA Flow Diagram.

The search strategy yielded 2579 results, which were reduced to 1995 once duplicates were removed. A total of 1921 records were excluded following title and abstract screening. Following full-text review of 74 records, only one study was deemed eligible and included in this review. Reasons for exclusion at the full-text screening stage included the following: wrong study design, for example, commentary pieces or qualitative studies on the management of COPD in care homes; wrong intervention, for example, COPD management interventions such as inhaler techniques; wrong outcome, for example, educational interventions for care home staff on other areas such as palliative care; and wrong population or setting, for example, education for hospital staff, patients, and/or caregivers. The screening and study identification process is presented in a PRISMA-ScR flowchart (Figure 1).

### 3.1. Characteristics of Included Studies

The one included study was a mixed methods study published in 2022 [44]. Further study characteristics are reported in Table 1.

### 3.2. Summary of Results

The intervention group generally rated the experience as a well-structured, good support. Prioritisation of the programme was difficult during their working day, with several participants advising that they completed it at home in their spare time. Participants stated that all modules were important to acquire knowledge about roles and healthcare services, but rated modules regarding ‘COPD and common problems’ most important and ‘COPD and follow-ups’ least important. The programme gave participants a desire to learn more, complete further courses, and encourage colleagues to complete it. Participants wanted to retain access to the COPD web and found it could be used for patient and peer education.

Pre-intervention interviews revealed that participants were not always informed of a patient’s diagnosis of COPD, and national guidelines for COPD were not routinely implemented in practice. Quantitative data revealed a statistically significant increase from 69% to 89% in objective COPD-related knowledge in the intervention group from pre- to post-test levels. Additionally, knowledge of the national guidelines showed a statistically significant increase from 64% to 80%. Qualitative data supported these knowledge increases with staff feeling more secure, and an increased attentiveness to COPD. Participants discussed an increased awareness of infections and the risks they pose, and an increased knowledge in breathing techniques, physical activity, signs of exacerbations, weight loss/nutrition, and diagnostics.

Although formal quality appraisal is not required within scoping reviews, due to the near-empty nature of the present review, some critical reflection is provided to aid interpretation. The feasibility trial component of the included study applied an a priori sample size calculation, validated measures, and appropriate statistical tests. Randomisation at the level of two municipalities and provision of tablets and email reminders added practical feasibility. The integration of data from the different phases of the study using a weaving approach allowed quantitative changes in knowledge to be interpreted alongside qualitative accounts of confidence, feasibility, and barriers to using the COPD web programme. However, the study’s small, predominantly female, convenience sample, limited description of specific care settings and professional roles, and lack of explicit discussion of data saturation or information power constrain generalisability to the wider care home workforce. Regarding setting, having been conducted in two Swedish municipalities within a publicly funded long-term care system, the findings may not readily transfer to the mixed public–private care home systems in many other countries.

## 4. Discussion

COPD is one of the most common and deadliest chronic diseases worldwide, requiring ongoing treatment, and in many cases necessitating long-term care placement due to a progressive loss of independence [45,46]. Despite COPD’s significant impact on patient health and healthcare systems, educational interventions regarding COPD management in care homes remains severely under-researched. This is reflected in this near-empty review. The comprehensive analysis in the present review identified only one eligible study on this topic, highlighting a major evidence gap in the development and evaluation of COPD educational interventions in care home settings. Empty and near-empty reviews play an important role by drawing attention to significant gaps in knowledge and highlighting areas that require further investigation [47,48]. The near-empty result of the present review underscores a critical need for further research, and this review provides a foundation for targeted recommendations for such future research and educational interventions.

Evidence from community settings has demonstrated that educational and self-management interventions for COPD can reduce hospital re-admission rates and improve patient outcomes [49]. Such interventions often included telemonitoring, education, empowerment for self-management, rehabilitation, multi-disciplinary teamwork, and post-discharge follow-up [49]. Yet, such interventions have not been adapted to care homes. This may stem from the persistent undervaluation of older adult care, particularly in care homes where care staff are often poorly compensated and older people are sometimes perceived as a drain on healthcare resources [50,51,52,53].

Although care home nurses play a critical role in promoting continuity of care, research into their perspectives and experiences with COPD management in care homes remains scarce. In other contexts, the few existing studies on continuity of care in palliative contexts also suggest a lack of targeted educational resources, as well as limited support for the emotional burden experienced by care home staff [54]. The broader literature on chronic disease management in care homes is primarily focused on dementia, although with promising outcomes [55]. For example, educational interventions in care homes may contribute to reduced behavioural and psychological symptoms of dementia [37,56,57,58]. In other areas, educational interventions have shown increased staff knowledge and confidence in the management of heart failure [38] and diabetes [59] within care home settings. Although these successful approaches do not involve COPD-related education, they act as illustrative examples of what targeted education could achieve in care home settings and call for similar application in COPD care, especially as the included study within the present review highlighted the feasibility and value of a digital COPD-related education resource in care homes [44]. However, the authors acknowledged the necessity for broader organisational change to embed new working practices, and thus, further research into these measures should be conducted [44].

Significant barriers limit the implementation and sustainability of educational interventions for COPD across other settings, which likely also contribute to the lack of COPD-related education in care homes. Reviews exploring the feasibility of chronic disease management interventions in primary care identified persistent concerns with the methodological rigour and sustainability of the studies, highlighting the difficulty of implementing effective education in care homes [60,61,62,63,64]. In care homes, such barriers may be exacerbated by factors such as high staff turnover, with reported rates as high as 56.2% for registered nurses and 78.1% for nursing assistants [65]. This instability can negatively impact efforts to invest in or sustain interventions for care home staff, especially time-consuming educational interventions. However, evidence from dementia training initiatives has suggested that comprehensive training programmes can help reduce staff turnover, likely by improving staff satisfaction and reducing stress [57,66]. Therefore, although implementation of these interventions may be challenging, their potential to produce broader positive effects on staff retention and care quality should not be overlooked.

The lack of training and educational resources for COPD management is especially notable considering expert consensus has already ranked the need to ‘identify optimal approaches to training’ as a key research priority to help reduce the global burden of COPD [67]. Priorities also called for further research on improved screening and accurate diagnostic methods for COPD in low-resource primary care settings. The discussed barriers may account for the lack of research addressing these priorities, while the included study in this review re-iterates these implementation challenges. Organisational barriers to the implementation of the digital educational resource were identified, including time constraints and insufficient programme support, challenges in interprofessional collaboration, limited health promotion services, and discrepancies between treatment guidelines and current practices [44]. These findings echo wider evidence that barriers to nurses’ continuing professional development, such as a lack of cover, using personal time or unpaid leave to complete training, and prioritisation of immediate, task-based responsibilities, are persistent and difficult to overcome [68,69,70].

Addressing organisational barriers is, therefore, crucial. Successfully improving COPD management in care homes will depend not only on bridging clinical knowledge gaps, but also on transforming embedded practices and workplace cultures. Without systemic change, even well-designed educational programmes may fail to deliver sustainable improvements. Removing these barriers will empower nurses, elevate care standards for residents, and foster a positive culture of ongoing education within care homes. The included study has provided evidence that COPD-specific education may increase staff knowledge and confidence [44]. Together with the broader literature, this suggests that care home staff education has the potential to improve chronic-disease management, but for COPD specifically, the evidence remains sparse. Further studies are needed before firm conclusions about the impacts of such interventions can be drawn.

### 4.1. Implications for Future Research and Practice

Given the lack of evaluated COPD-related education for care home staff identified in this review, future research should prioritise the development of curricula that address core content areas directly relevant to everyday practice. These include early recognition and management of exacerbations, appropriate escalation and communication with primary and emergency care, optimisation of inhaler technique and oxygen use, symptom management, nutrition, and advanced care planning [71]. Given emerging evidence that combining nutrition therapy and rehabilitation can preserve muscle mass and improve functional outcomes following COPD exacerbations [19,21], COPD education for care home staff should explicitly incorporate content on recognising and managing malnutrition, the role of physical activity, and simple rehabilitation strategies in long-term care [16,17,22]. Additionally, information on timely referral pathways and collaboration with dietitians and physiotherapists may help to facilitate early mobilisation and practical ways to support dietary intake and exercise within resource-constrained environments. Incorporating education on interprofessional working and role clarity between nurses, care assistants, and allied health professionals is also likely important, given the multidisciplinary nature of long-term care.

The included study suggests that digital, self-directed learning can improve knowledge but may need additional organisational support and facilitation to ensure uptake in busy care home environments [44]. Future interventions could therefore test blended models that combine flexible web-based modules with brief face-to-face or virtual workshops, peer discussion, and clinical facilitation to support translation into practice [72,73,74]. The co-design of such programmes with care home staff and management, as well as residents and family members, may also help tailor the content and delivery to local constraints and preferences.

Future studies should incorporate outcome measures to explore impacts at the staff, resident, and service level, for example, COPD-specific knowledge measures for staff, as well as measures of confidence and self-efficacy in COPD care, while, for residents, symptom burden and health-related quality of life outcomes would add to the evidence base. Finally, at a service level, key outcomes may include the numbers of emergency hospital visits and unplanned GP appointments. Reporting such outcomes will directly address the gaps highlighted by this near-empty review and enable future reviews to move beyond mapping towards robust assessments of effectiveness.

### 4.2. Strengths and Limitations

The present review applied a rigorous, systematic approach to mapping existing evidence on COPD-related education for care home staff, yet identified only one eligible study in the area. This near-empty result means that conclusions draw on a single study, but also clearly signals a profound evidence gap in the evaluation of COPD educational interventions within care home settings. This review, therefore, offers a valuable contribution by making explicit the scarcity of evaluated interventions and by outlining key avenues for future intervention development and testing in this field.

This review followed internationally recognised methodological and reporting guidance for scoping reviews, including a pre-specified protocol, and a comprehensive search strategy across multiple databases. However, some limitations should be acknowledged, including the inclusion of only studies in the English language, and the exclusion of grey literature such as policy reports and other non-peer-reviewed evidence. These restrictions may have led to the relevant literature not in the English language, or unpublished evidence, being missed. Grey literature was not systematically searched, in order to focus on peer-reviewed studies. However, in the context of care home education, it may be likely that initiatives are developed and evaluated locally without progressing to journal publication, which may have contributed to the near-empty outcome of this review. This could limit the completeness of the evidence map and the extent to which the findings capture real-world implementation practices in diverse care home contexts. Future evidence synthesis on COPD education may benefit from consideration of a broader approach which incorporates grey literature.

## 5. Conclusions

In conclusion, this scoping review demonstrates that, despite the high burden of COPD among care home residents, and the central role of staff in recognising and managing symptoms, there is almost no empirically evaluated education or training available. The identification of only one eligible intervention highlights a critical missed opportunity to strengthen nurses’ and care assistants’ knowledge, confidence, and skills in COPD management. Addressing this gap has clear implications for nursing practice, ultimately enhancing the quality of care, and the quality of life, for those with COPD in care home settings.

Future studies should prioritise co-design, implementation, and robust evaluation of comprehensive COPD educational programmes tailored to care home staff. Practice-relevant outcomes such as staff knowledge and behaviour, rates of hospital admissions, and residents’ quality of life and symptom burden should be explored. By generating such evidence, consistent and effective COPD management within care homes may be improved.

Overall, this scoping review serves as a foundation for future research into COPD education in care homes. By highlighting the lack of evaluated interventions, future research and quality improvement initiatives may build on the strengths of the existing study and the recommendations provided. Any future interventions provide an opportunity for meaningful advancement in practice and care quality.

## Figures and Tables

**Table 1:** Characteristics of included studies.

Author (Year), Country	Research Aim	Study Methods	Sample and Setting	Key Findings
Nyberg et al. (2022) [44], Sweden	To evaluate a digital COPD education programme for healthcare professionals in care homes regarding feasibility, knowledge, and working procedures. The intervention consisted of a digital COPD program delivered through an interactive web-based platform called the COPD Web. This included six themed modules which took 20–30 min each to complete. Participants were provided a tablet and internet access for 12 weeks to access the programme.	Convergent mixed methods design including a randomised controlled feasibility trial and repeated semi-structured interviews. Education programme was accessible for three months and data was collected via questionnaires on COPD-specific knowledge (Bristol COPD Knowledge Questionnaire), conceptual knowledge, feasibility, and usage of the COPD web platform. Repeated individual semi-structured interviews were performed; one before the intervention period and one after the three-month period. Quantitative and qualitative findings were merged.	Data collection occurred between January and May 2018. Convenience sampling of registered healthcare professionals working in two municipalities in the most northern county councils in Sweden. The sample included 18 care home nurses, 10 occupational therapists, 8 physical therapists, and 1 dietitian. 37 participants completed the trial (86% female): 20 participants in intervention group, and 17 in control group. 11 participants completed both interviews.	The COPD educational programme appeared feasible based on the satisfaction of the sample and reports that it had supported their work. Objective COPD-specific knowledge increased. Care home nurses described feeling supported by the intervention and liked the structure. Suggestions for improvement included adding more movies, pictures, and recorded lectures in combination with the text. Participants expressed a desire for further training about COPD and encouraged colleagues to complete this training.

## Data Availability

No new data were created or analyzed in this study.

## Supplementary Materials

The following supporting information (Search Strategy, PRISMA-ScR Checklist) can be downloaded at: https://www.mdpi.com/article/10.3390/nursrep16020043/s1. Supplementary Material 1: PRISMA-ScR Checklist; Supplementary Material 2: Search Strategy.

## Public Involvement Statement

No public involvement in any aspect of this research.

## Guidelines and Standards Statement

This manuscript was drafted against the Preferred Reporting Items for Systematic Reviews and Meta-Analyses extension for Scoping Reviews (PRISMA-ScR) Checklist.

## Abbreviations

The following abbreviations are used in this manuscript:

COPD	Chronic Obstructive Pulmonary Disease
JBI	Joanna Briggs Institute
PRISMA-ScR	Preferred Reporting Items for Systematic Reviews and Meta-Analyses extension for Scoping Review
QoL	Quality of Life
MSc	Master of Science
NMC	Nursing and Midwifery Council
